# THE ROLE OF THE ENDOPLASMIC RETICULUM UNFOLDED PROTEIN STRESS RESPONSES IN A C. ELEGANS MODEL OF QUIESCENCE

**DOI:** 10.1093/geroni/igac059.2674

**Published:** 2022-12-20

**Authors:** Arianna Pankey

**Affiliations:** Howard University, Washington, District of Columbia, United States

## Abstract

Cells devote extraordinary energy to translate and fold approximately 20,000 proteins encoded by the human genome. About one third of these proteins are transmembrane proteins—correctly folded in the endoplasmic reticulum (ER). The ER Unfolded Protein Response (UPRER) is a response to disturbances at the ER. As an organism ages, cells lose the ability to activate the UPRER. Our lab uses C. elegans—a microscopic roundworm—as a model organism to determine how loss of ER function can contribute to aging. The UPRER plays a key role in maintenance of functional quiescence. Quiescent cells exist in a reversible state of dormancy and can receive specific environmental cues to proliferate. The developmental state known as L1 arrest resembles the same features as quiescent cells. IRE-1 is required for worms to exit their quiescent state after starvation. Understanding the genetic pathways that are best at suppressing ire-1 terminal L1 arrest could indicate ways to improve functional quiescence. We forced the worms into an L1 arrest state; The unfolded protein response in the endoplasmic reticulum maintains functional quiescence within the cell. The worm’s ability to develop once they exit this quiescent stage effectively, indicated the ability of the worm to maintain protein homeostasis despite non-ideal conditions. It was found that all tested mutations were unable to demonstrate improvement in ability to maintain development when arrested long-term. Our data indicates that our worms were counted prematurely. In the future, we will allow for a longer growth period as well as larger quantities of food.

